# A Report from the Cambodia Training Event for Awareness of Melioidosis (C-TEAM), October 2017

**DOI:** 10.3390/tropicalmed3010023

**Published:** 2018-02-23

**Authors:** Sotharith Bory, Frances Daily, Gaetan Khim, Joanne Letchford, Srun Sok, Hero Kol, Muy Seang Lak, Luciano Tuseo, Chan Vibol, Sopheap Oeng, Paul Turner

**Affiliations:** 1 Infectious Diseases Unit, Calmette Hospital, Phnom Penh 12201, Cambodia; sotharith_bory@yahoo.com; 2 Diagnostic Microbiology Development Program, Phnom Penh 12302, Cambodia; gaetan.khim@dmdp.org (G.K.); joanne.letchford@dmdp.org (J.L.); oeng.sopheap@dmdp.org (S.O.); 3 Hospital Services Department, Ministry of Health, Phnom Penh 12152, Cambodia; soksrun@gmail.com; 4 Preventive Medicine Department, Ministry of Health, Phnom Penh 12152, Cambodia; herokol@yahoo.com (H.K.); sreanglak@yahoo.com (M.S.L.); 5 World Health Organization, Phnom Penh 12302, Cambodia; tuseol@who.int (L.T.); chanv@who.int (C.V.); 6 Cambodia Oxford Medical Research Unit, Angkor Hospital for Children, Siem Reap 17252, Cambodia; pault@tropmedres.ac; 7 Centre for Tropical Medicine and Global Health, Nuffield Department of Medicine, University of Oxford, Oxford OX3 7FZ, UK

**Keywords:** melioidosis, Cambodia, epidemiology

## Abstract

Melioidosis is an endemic infection in Cambodia, a lower middle income SE Asian country. Despite more laboratories isolating and identifying *Burkholderia pseudomallei* in recent years, the infection remains under-recognised and under-diagnosed, particularly in the adult population. Lack of knowledge about the disease and lack of utilization of microbiology laboratories contributes to this, along with laboratory capacity issues. Treatment costs often hamper optimal management. In response to these issues, a national one-health training event was held in October 2017 to raise awareness of the disease amongst clinical, laboratory, and public health professionals. The meeting format, findings, and outcomes are described here.

## 1. Introduction

Melioidosis, infection by the environmental Gram-negative bacterium *Burkholderia pseudomallei*, is an endemic but significantly unrecognized disease in Cambodia, a lower middle income SE Asian country with a population of ~16 million. It was first diagnosed in Phnom Penh in 1928 in a Russian national, although he was almost certainly infected in Thailand [1]. Subsequently, pulmonary melioidosis was described in a resettled refugee who had lived in Thailand for several years before diagnosis [2], and also a porcine outbreak was identified in the 1960s [3]. However, it was not until 2005, that human melioidosis cases began to be regularly identified in-country, initially in children following the establishment of a diagnostic microbiology laboratory at Angkor Hospital for Children, Siem Reap [4]. Since then, significant laboratory capacity building has occurred nationally and several hundred cases in both children and adults have been described [5,6,7,8,9]. Not unexpectedly, mortality is higher in adults (more than 50%) than in children [4,6,7,8]; however, as with adults, children who are bacteraemic have higher mortality (72%) [8]. Serologic screening using the indirect haemagglutination assay (IHA) revealed that 16% of tested children from Siem Reap province had evidence of exposure to *B. pseudomallei* and the organism was confirmed to be present in rice paddy soil [10].

A recent global mathematical modelling study predicted that in 2015, Cambodia would have had 2083 (95% credible interval 850–5451) melioidosis cases resulting in 1149 (464–3042) deaths [11]. Lack of clinician awareness and limited diagnostic microbiology capacity may explain the discrepancy between the model predictions and the low number of cases confirmed in Cambodia each year.

With aim of improving clinical, laboratory, and public health professional awareness of melioidosis, a national one-health training event was held in Phnom Penh, 17–19 October 2017.

## 2. The C-TEAM Meeting

There were more than 180 meeting attendees, representing national, provincial and non-governmental hospitals, universities and research institutes, as well as government ministries (Health, Agriculture, Fisheries and Forestry, Environment, Rural Development), international and non-governmental organizations and partners. Hospitals were asked to prepare a summary of melioidosis cases and *B. pseudomallei* isolate numbers prior to the meeting. Where possible, it was requested that data be stratified by date (year), specimen type, and geo-location (home province). Prior to presentation every effort was made to verify the accuracy of the data.

The first day featured plenary talks from international clinical and laboratory melioidosis experts, followed by situation updates from several national, provincial and non-governmental hospitals. Representatives from six major participating hospitals presented data on their clinical and laboratory capacity. In particular, melioidosis case numbers, diagnostic procedures, and treatment regimens were shared. The day concluded with presentations on melioidosis in animals, environmental reservoirs of *B. pseudomallei*, prevention and public engagement activities.

The second and third days featured parallel workshop sessions for clinicians and laboratorians. Cambodian clinicians presented cases to demonstrate the breadth of melioidosis infection, including pneumonia, sepsis, head and neck abscesses, hepatic, splenic, prostatic abscesses, and bone and joint infections. Expert speakers provided commentary on the cases and led discussions around diagnosis and treatment. Particular attention was paid to defining the optimal diagnostic approach and appropriate treatment choices [12,13]. Sessions on the radiologic features and surgical management of melioidosis were included. The recently updated national treatment guidelines were presented and discussed and encouragement was given to participants to collect case data prospectively in the hope of gaining more knowledge about the local epidemiology. Laboratorians gave local situation updates and participated in dry and wet laboratory sessions to gain experience of best practices for safe handling and identification of *B. pseudomallei* from clinical specimens, as well as determination and reporting of appropriate antimicrobial susceptibilities. Identification test demonstrations and hands-on practical sessions included the three-disk test (co-amoxiclav, colistin/polymixin B, and gentamicin) [14], InBios Active Melioidosis Detect lateral flow assay [15], and Mahidol University latex agglutination test [16,17]. There was a demonstration of the advantages of Ashdown’s agar and broth, *B. pseudomallei* selective media, for culture of non-sterile site specimens such as throat swabs [18]. Diagnostic Microbiology Development Program (DMDP)-developed standard operating procedures and job aids were shared with meeting participants. Two training videos were prepared for the meeting, providing overviews of specimen processing and identification of *B. pseudomallei* (the Khmer language version can be found at: https://vimeo.com/237880199).

The meeting concluded with an interactive session for clinicians and laboratorians aimed at promoting communication and identification of areas where co-operative efforts between clinical and laboratory teams could be used to improve diagnosis and management patients with melioidosis.

## 3. Summary of the Current Situation

**Figure 1:**
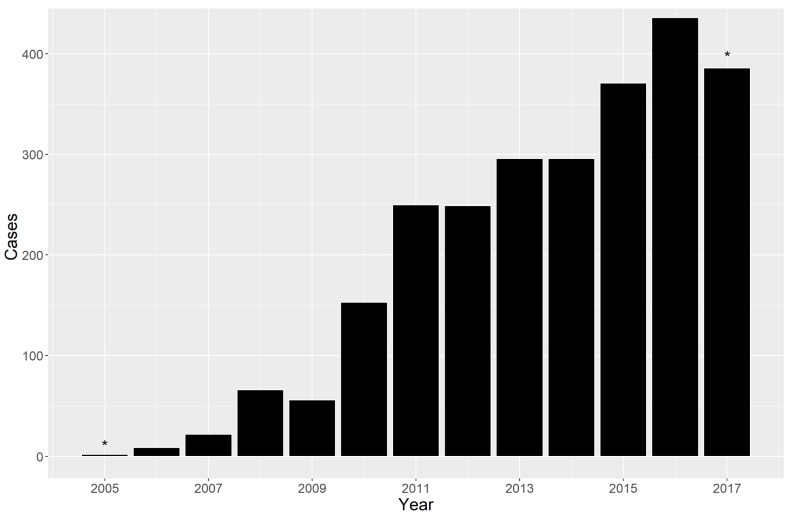
Annual numbers of confirmed melioidosis cases in Cambodia, October 2005–September 2017 (* incomplete years).

Since the first national melioidosis conference, held in August 2010, there has been a steady increase in the number of culture confirmed cases from 173 (isolated and identified from five microbiology laboratories since October 2005) to 2592 up to September 2017 (from 17 microbiology laboratories) (Figure 1). Partner organizations, including the DMDP, have worked with the Ministry of Health Bureau of Medical Laboratory Services to improve laboratory diagnostic capacity over this time. Despite the increase, the annual cases remain well below the levels predicted by the mathematical model.

To date, the majority of cases have been children (at least 60% (1565/2592), but age data not available for all cases), which is in contrast to other countries where children account for 5–15% of all cases. This is likely due to overrepresentation of cases from three children’s hospitals that have well-established microbiology laboratories and clinical diagnostic algorithms. In many hospitals, both public and private, there is as yet no systematic sampling for patients with presumed infections, hence very few microbiology investigations are requested by doctors. In addition, in many hospitals in Cambodia, some patients are required to pay for services including microbiology testing and treatment, which is often cited as a barrier to increasing the number of specimens requested and treating the patient with recommended treatment.

**Figure 2:**
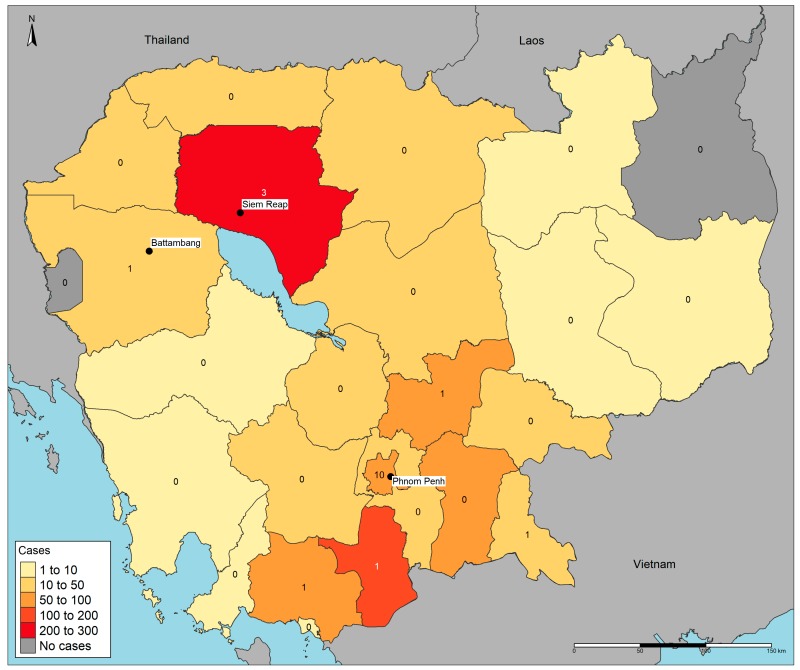
Geographic distribution of melioidosis cases in Cambodia, October 2005–September 2017. Province of residence was available in 889 confirmed cases. Shading represents the total number of culture-confirmed melioidosis cases per province. The numbers represent the count of participating microbiology laboratories per province; however, one was unable to contribute its culture confirmed cases prior to the meeting.

Data on culture-confirmed *B. pseudomallei* cases are not yet routinely collated at a national level. Of data obtainable and presented at the meeting, home province information was only available in one third of patients (34%, 889/2592). Despite this, the data confirm that 23 of 25 provinces have had culture-confirmed cases, suggesting the infection is endemic throughout the country (Figure 2). As soil testing has been limited to one province and no water studies have yet been carried out, it is unknown whether there are areas in the country where the infection is more likely to be acquired.

The month of culture confirmation was available for only one fifth of all cases, but where recorded, the majority of cases (71%, 377/528) occurred during the wet season months of May to October, which is consistent with other endemic areas. Where documented (19% of all cases; 481/2592), adults were more likely to have risk factors than children, with the most common being diabetes mellitus, hazardous alcohol use and corticosteroids. The majority of adults had presented with pneumonia and/or sepsis, whereas children were more likely to have head and neck infections. Both of these findings are consistent with other endemic countries; however, one province had a high number of head and neck presentations in adults (30% of all presentations), which is unusual in this age group.

The most common *B. pseudomallei* culture-positive specimen type reported was pus (55%, 1530/2765 specimens) followed by blood culture (34%, 941/765). Only 73 sputum cultures were documented, despite pneumonia being a common clinical presentation. Bronchoalveolar lavage/aspirates were positive in 17 specimens and there were 26 pleural fluid specimens positive for the bacterium. Commonest diagnostic methods in routine use were the three-disc test (82%, 14/17 laboratories) and the bioMerieux API 20NE test strip (59%, 10/17 laboratories). One third (35%, 6/17) of the laboratories reported use of Ashdown selective media, although it is not yet routinely used or readily available in all of these laboratories. Several laboratories reported routinely releasing antibiotic susceptibility data on drugs that are not recommended for melioidosis treatment, such as ceftriaxone, cefuroxime, colistin and fosfomycin. Prior to the meeting, some laboratories were unaware of the limitations of disc diffusion testing for co-trimoxazole [19]. 

Treatment choice and length of treatment varied between hospitals, with some using ceftriaxone during the initial phase, rather than the recommended ceftazidime. Many hospitals reported they often lack sufficient supplies of ceftazidime and therefore treat patients with inferior drugs (e.g., co-trimoxazole or ceftriaxone) or with shortened courses of ceftazidime. Carbapenems are not readily available in almost all hospitals, so rarely prescribed. Eradication phase options sometimes included co-amoxiclav as first-line treatment, rather than the recommended co-trimoxazole, mostly without consideration of amoxicillin:clavulanate ratio. The length of treatment and follow up during this phase was inconsistent. Outcome data was very incomplete with hospitals often having no information as patients were lost to follow up, or outcome had not been collated.

## 4. Outcomes and Future Plans

The major outcome of the meeting was the successful drawing together of a broad range of Cambodian health professionals to share knowledge and experience of the epidemiology, clinical and laboratory diagnosis, treatment and prevention of melioidosis. Many of the animal and environmental sector participants heard for the first time about the importance of this disease and were eager to learn more. Local data were collated and presented alongside state-of-the-art lectures from global melioidosis experts. Current challenges in clinical case detection, awareness of risk factors (e.g., diabetes), laboratory diagnosis, treatment and limited collation of epidemiological data were highlighted. Updated national diagnosis and treatment guidelines were showcased. Laboratory staff were made aware of current best practices for safe culture and identification of *B. pseudomallei,* and were provided with key standard operating procedures and job aids. The need for reliable surveillance data was recognised as a priority. It is hoped that the enthusiasm shown by all participants at the meeting will be translated into a sustained effort to improve clinical diagnosis, laboratory confirmation, treatment, outcomes and surveillance of melioidosis in Cambodia moving forwards. Further training workshops, plus development and provision of an information package summarizing the current situation and outstanding challenges to policy makers, clinicians, laboratory technicians and other stakeholders, will consolidate the considerable momentum generated at the C-TEAM meeting.
